# Implementation and evaluation of a specialized diabetes clinic in Guinea-Bissau: lessons learnt from the field

**DOI:** 10.11604/pamj.2020.37.126.26127

**Published:** 2020-10-05

**Authors:** Jorge César Correia, Adalgisa Lopes, Adramane Nhabali, Victor Madrigal, Carlos Reguera Errasti, Emer Brady, Michelle Hadjiconstantinou, Montserrat Castellsague Perolini

**Affiliations:** 1 Unit of Patient Education, Division of Endocrinology, Diabetology, Nutrition and Patient Education, WHO Collaborating Center, Department of Medicine, Geneva University Hospitals and University of Geneva, Geneva, Switzerland,; 2 Association Suisse d´Aide aux Personnes Diabétiques en Guiné-Bissau, Geneva, Switzerland,; 3 Department of Internal Medicine, Hospital Nacional Simão Mendes, Bissau, Guinea-Bissau,; 4 Aida Ayuda Intercambio y Desarrollo (AIDA), Bissau, Guinea-Bissau,; 5 Leicester Diabetes Centre, University Hospitals of Leicester, NHS Trust, UK,; 6 Division of Tropical and Humanitarian Medicine, Geneva University Hospitals, Geneva, Switzerland

**Keywords:** Diabetes mellitus, Guinea-Bissau, training programs, consultation clinics

## Abstract

**Introduction:**

diabetes care in Guinea-Bissau (GB) is characterized by a lack of properly trained healthcare professionals (HCPs) and guidelines for diagnosis, treatment and follow up of patients. To address these issues, this project was launched with the objective to train HCPs in the management of diabetic patients and establish a specialized diabetes clinic in the Hospital Nacional Simão Mendes, a public tertiary care hospital in Bissau, capital of GB. This project is led by the Geneva University Hospitals (HUG) in collaboration with the Swiss Association for the Aid to Diabetic People in Guinea-Bissau, with the support of the International Solidairty Office (SSI) of the State of Geneva, and AIDA (Ayuda, Intercambio y Desarrollo).

**Methods:**

specialists from the HUG in collaboration with local experts in GB developed and delivered a culturally and contextually adapted training course pertaining to diabetes care to HCPs in this hospital. Pre and post training tests were conducted to assess differences in knowledge and practices. Following the training program, a diabetes clinic was set up and an audit was conducted to assess its performance.

**Results:**

a total of 24 HCP attended the training program and exhibited statistically significant improvements in their knowledge pertaining to diabetes care (mean difference between pre and post-test = 14.53, SD 11.60, t=-4.8, p < 0.001). The diabetes clinic was established and provided consultations 2 days per week. A total of 63 patients consulted at this clinic, of which 49 had type two diabetes treated with oral antidiabetic drugs and 14 were type 1 diabetics treated with insulin. Patients had blood glucose measurements and received therapeutic, dietary and physical activity counselling. Several barriers leading to occasional interruptions of service were encountered, including a political instability in the country and strikes of healthcare staff demanding better wages and working conditions.

**Conclusion:**

this study delineates the feasibility of setting up a diabetes consultation clinic in GB despite important barriers. To ensure successful running of such consultation clinics, continued buy-in and support from stakeholders should be ensured. Diabetes training should be incorporated in pre-and post-graduate training curriculums of all HCP to help shape a better workforce.

## Introduction

Diabetes mellitus (DM) is a global challenge due to its growing incidence and associated long-term complications and poor quality of life. The global prevalence of DM among adults has risen from 4.7% in 1980 to 8.5% in 2014, with a higher preponderance toward low- and middle-income countries (LMIC) [1]. The International Diabetes Federation (IDF) estimated that 382 million adults had DM, causing 5.1 million deaths in 2013, and the burden is expected to increase [2]. Especially in Sub-Saharan Africa (SSA), a recent dramatic change in age-related demographics as well as lifestyle changes is the leading factors of this rising prevalence [3-6]. This is also evident in the republic of Guinea-Bissau (GB), a country lacking in health care infrastructure, resources, and a well-integrated health system [7]. In GB, precise estimates for the prevalence of DM are not available, owing to a lack of research studies or surveys of high standards. According to available studies conducted in selected populations in GB, the prevalence of DM ranges from 1.7 % up to 14.3% [8-10]. This prevalence is lower than those reported in global and regional prevalence surveys [2,11,12]. However, the current prevalence estimates are considered to represent only the tip of the iceberg, due to the important number of undiagnosed cases. This was reflected in the survey conducted by Haraldsdottir *et al*. where none of the diagnosed people with DM (PWD) were registered at the local diabetes clinic, and were unaware of their impaired glucose level status [8]. According to Byberg *et al*. the prevalence of undiagnosed diabetes is estimated to be 9% [10].

This situation led researchers to call for the implementation of prevention and early diagnosis measures for DM in GB, to avoid burdening the local fragile health system [9,10]. In this context, we conducted a situational analysis of DM care in GB to identify main issues faced in DM management in the country [13]. This qualitative study revealed several major barriers, including the lack of specialist diabetic care in the region, and poor standardized procedures for diagnosis, treatment and follow up of patients [13]. To address these issues, the present project was launched as the first concerted effort, to train health care professionals (HCPs) in the management of diabetic patients and establish a specialized diabetes clinic in a Hospital in GB. This project was launched by the Geneva University Hospitals (HUG) in collaboration with the Swiss Association for the Aid to Diabetic People in Guinea-Bissau, with the support of the International Solidairty Office (SSI) of the State of Geneva, and AIDA (Ayuda, Intercambio y Desarrollo), a Spanish non-governmental organization (NGO). This paper reports the implementation process as well as the evaluation of activities.

## Methods

**Figure 1:**
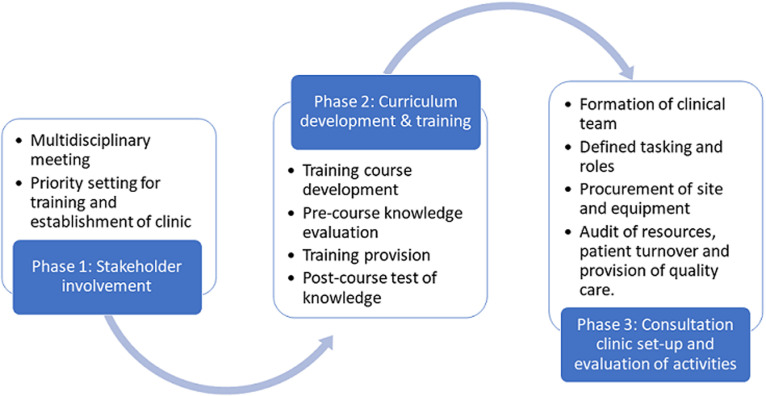
flow diagram for study process

This study was conducted in the Hospital Nacional Simão Mendes (HNSM), a public sector, tertiary care hospital in Bissau, the capital of GB. This hospital is the only tertiary care hospital in the region, with over 500 beds and houses several medical and surgical specialties. However, there is no endocrinologist and there is a general lack of knowledge regarding diabetes care in the hospital. The project consisted of three sequential phases describes below and illustrated in Figure 1.

**Figure 2:**
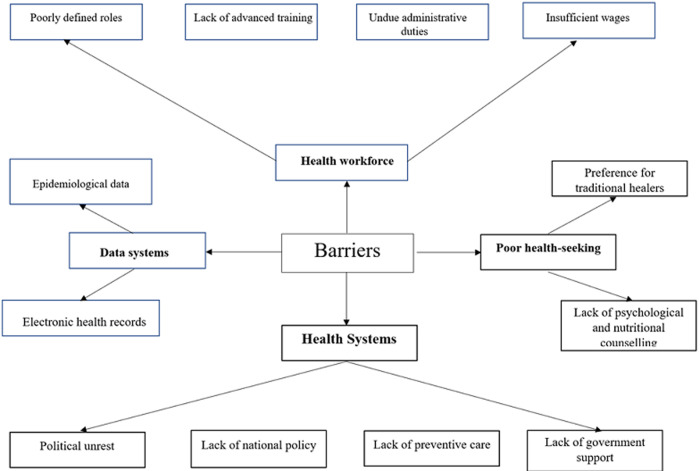
barriers to foundation of diabetes clinic in Guinea-Bissau

**Phase 1 (stakeholder involvement):** following the situational analysis [11], a meeting was held between study investigators and 15 multidisciplinary healthcare professionals and stakeholders based in GB. These participants included the deputy clinical director of the HNSM and specialist physicians (n=5) involved in the care of PWD, with such specialties as internal medicine (n=2), emergency medicine (n=1), pediatrics (n=1) and orthopedic surgery (n=1). Also participated in the meeting nutritionists (n=4), a member of the Ministry of Health and social workers representatives of non-governmental organization working for care of PWD (n=2). In this interdisciplinary meeting, the main issues regarding diabetes care in the HNSM were discussed including i) lack of defined roles at the hospital ii) lack of staff training and multidisciplinary discussions iii) undue administrative duties of clinical staff iv) instability and lack of coherence in national policy making and clinical efforts at the hospital v) lack of motivation in using electronic health record systems vi) insufficient wages vii) lack of transport viii) lack of government support ix) lack of access to healthcare x) lack of psychological and diet counselling support to the patients and xi) lack of epidemiological data to inform policy making and resource allocation (Figure 2). Thereafter, to improve the delivery of diabetes care in HNSM, specific action points were formulated which included the development of a specialized consultation for PWD in the HNSM with a mobile multi-professional team (i.e. doctors, nurses, nutritionists, social workers), that would also support other HNSM units regarding care of PWD. A plan was devised to involve several NGOs to help provide services to those most in need, including ASLUCO Diabetes GB (Associação de luta contra a diabetes em Guinée-Bissau) and AIDA (Ayuda, Intercambio y Desarrollo) that help with the free provision of medication for people with limited resources using assessment by social workers. Long term goals include creating synergies between governmental and non-governmental organizations that are working in isolated capacities in GB, in order to unite strengths and expertise, inspire advocacy and solicit government support.

**Phase 2 (knowledge sharing, curriculum development and training):** to develop the diabetes training program curriculum, and ensure the grounding of the course within the resources available in the country, preparatory discussions were held between the specialists from the HUG and HCPs from the HNSM. It was decided that the HUG faculty would develop presentations on the international recommendations in diabetes care with a local HCP who would discuss the adaptation of these recommendations according to the human and material resources available in the hospital. Inclusion of local clinicians along with faculty from HUG, in the delivery of course was reinforced. The Leicester Diabetes Center team was also contacted for provision of diabetes self-management educational tools adapted to the African context and translated into Portuguese through a similar project run in Mozambique. A detailed plan of the educational program is provided in Table 1. Pre and post training tests were conducted to assess differences in knowledge and practices of healthcare professional who attended the course. It included questions exploring the participants´ knowledge and practices pertaining to DM across several domains such as its etiology, diagnosis, medication, testing and prevention. The feasibility of this training program was evaluated using a questionnaire administered after the conduct of the training program. It contained a set of questions measured on a Likert Scale exploring one´s confidence in the management of different aspects of diabetes care. It included statements such as, “I am able to provide nutritional counselling to people with diabetes?” answered on a Likert scale format from strongly agree to strongly disagree. The questionnaire also contained open ended questions evaluating the course, including: a) Do you feel you have reached your expectations by participating in this course? Please, state reasons. b) Did you find the course interesting? c) Did you enjoy participating in the course? d) Did the methodology for conduct of course facilitate your learning? Please, explain. e) Any comments, proposals and suggestions for future activities. Quantitative variables were presented as mean (SD) and qualitative as frequency (%). Paired T-test was conducted to evaluate the statistical significance pertaining to differences in baseline vs post-training scores. Open-ended responses from the participants were analyzed using a narrative synthesis strategy.

**Table 1:** summary of training curriculum

Module	Delivery format
Knowledge assessment pre-training	Multiple choice questions
Type 1 and 2 diabetes: pathophysiology and risk factors	Power point presentation
Type 1 and 2 diabetes: pharmacologic approach	Power point presentation
Type 1 and 2 diabetes: nutrition	Power point presentation and practical demonstrations with fake props
Type 1 and 2 diabetes: physical activity	Power point presentation
How to monitor diabetes?	Blood glucose and injection techniques: power point presentation
Treatment and monitoring practical seminar	Hands-on workshops: - blood glucose measuring techniques - insulin injection and mixing techniques
Acute complications and their management: hypo, hyper, ketoacidosis/hyperosmolar coma	Power point presentation
Clinical seminar: management of chronic complications	Power point presentation
Clinical seminar	Interactive discussion from real clinical cases
Patient education and diabetes self-management education	Power point presentation and practical role playing exercise
Patient education and diabetes self-management education	Practical workshop: how to uncover the needs of each PWD?
Patient education and diabetes self-management education	Ambassador's technique: guide for interviewing patients and their families
Post-training evaluation	Repeat of the first day questionnaire

**Phase 3 (consultation clinic set-up and evaluation of activities):** a thorough discussion was conducted on practical aspects for setting up the consultation clinic for PWD. Duties for specific health professional were assigned and protocolized. Agreements were made on the wider activities that the nurses can competently undertake in the absence of physician supervision (history, weight, height, blood sugar and HbA1c measurement, assessment of eating habits and physical activity). The partner NGOs (AIDA, ASAPDGB and ASLUCO diabetes GB) advertised the opening of the clinic on the radio so that the population was made aware of the existence of this consultation. The logistical aspects of delivering a successful consultation were discussed and decided upon. Equipment was provided including devices to measure the blood glucose (glucometers, lancets, strips and glycated hemoglobin device) and anthropomorphic values (weight, height, waist circumference). Antidiabetic drugs and insulin were also provided. The establishment of the consultation clinic started on July 15, 2019. This outpatient consultation clinic was designed to run twice a week, managed by two doctors and a nurse, who also carried out consultations for PWD hospitalized in the various services of the HNSM. On a monthly basis, an audit of resources, patient turnover and provision of quality of care was assessed by the clinical director of the HNSM with feedback provided to the HUG team and partner NGOs, in order to discuss any adjustments that need to be made to improve the consultation.

## Results

**The diabetes training program:** the training program was held from 22^nd^ to 27^th^ June, 2019. There was a total of 24 trainees including doctors (n=13), nurses (n=8), nutritionists (n=2), and a representative from an NGO. The course was led by two experts from the HUG (one doctor and one nurse) with nine local experts as co-facilitators including internist (n=1), emergency physician (n=1), nephrologist (n=1) ophthalmologist (n=1), orthopedist (n=1), pediatricians (n=3) and dermatologist (n=1). Complete baseline and post-training scores were available for 15 participants, with a response rate of 62.5%. Mean score prior to training was 68.53 (18.39). Participants showed good knowledge of the signs, clinical conditions, and the notion that there are different types of diabetes. But there was a lack of clear understanding in the pathophysiological differences, glycemic references, and the bases of treatment. There were shortcomings in terms of patient monitoring (especially concerning use of glycated hemoglobin). There was a significant increase in post-test scores among the participants with a mean score of 83.07 (15.99) (mean difference= 14.53, SD 11.60, t=-4.8, p < 0.001). We note a favorable progression of all participants. The most significant progress was observed among nurses (61.78+12.00 to 82.66+13.94). The highest marks indicative of greater knowledge, however, persisted among doctors (87.0+6.94 to 96.4+8.47). Regarding the qualitative analysis, a total of five participants provided a detailed qualitative feedback using the open-ended questionnaire. All participants praised the course facilitators delivering the course in a simple language, and that the content was interesting and interactive. The participants commented that pre-course discussions to understand the local context and shortcomings, helped cover the participants´ gaps in knowledge and clinical management of the patients with diabetes. Use of logical and conceptual learning helped deepen basic concepts and knowledge and sharing of lived experience with the participants was particularly found to be helpful. An interactive learning environment with opportunities for participants to learn via participation was found to be helpful. Use of demonstrations and practicing with peers was also praised. In addition to better management, physicians reported feeling confident in tackling allied topics of dietetic education and recommendations for therapeutic physical exercise. They understood the importance on teaching methods of insulin shots and balanced diet to PWD. Moreover, use of insulin pumps was novel for the participants. They were confident that they would be more efficient and provide a better service to their patients. Two physicians recommended to expand the duration of future trainings. A suggestion was provided to include obstetricians as course facilitators to discuss the topic of gestational diabetes in more detail. Participants recommended to deliver this training several times and stressed upon foundation of a diabetic foot clinic. More efforts were solicited in consolidating the connections between doctors from different specialties such endocrinology and orthopedics to improve diabetic patient care.

**The diabetes consultation clinic:** following the training program, the diabetic consultation clinic was inaugurated in July 2019. This clinic was run two days a week by a team of 8 doctors and nurses who completed the training program and were identified by the hospital clinical director. The nutritionists, however, did not participate in these clinics with one migrating to another country and unavailability of the second. A total of 63 PWD consulted at this clinic, of which 49 had type two diabetes treated with oral antidiabetic drugs and 14 were type 1 diabetics treated with insulin. Of these patients, 11 people attended for screening purposes without previous knowledge of disease, of whom 2 were positive, meeting the criteria for diabetes. Among the PWD, only the blood glucose values of the first visit were measured, averaging 219.6 mg/dl (12.2 mmol/l) which shows a poor glycemic control. The glycated hemoglobin was performed in two patients confirming the poor overall glycemic control (8.0% and 8.3% respectively). Consultations included therapeutic, dietary and exercise counselling. Each patient was provided with glycemic measurement, weight control, and supply of metformin and insulin therapy as required. A summary of activities and patient characteristics are described in Table 2. The functioning of the clinic and patient follow-up was adversely affected by several barriers including an important political instability in the country and frequent strikes by hospital staff to demand better wages and working conditions. This led to frequent interruptions of service of the clinic.

**Table 2:** HNSM diabetes clinic overview from July to September 2019

Nature of visit				Diabetes type		Gender		Glycemic control (averages)	
1^st^ consultation	1^st^ follow-up	2^nd^ follow-up	3^rd^ follow-up	Type 1 diabetes	Type 2 diabetes	Male	Female	Random plasma glucose test	HbA1c
63	10	37	11	14	49	28	35	219.6 mg/dl	8.15%

## Discussion

We have successfully trained a multidisciplinary team of healthcare professionals in the basic management and care of PWD and set up a specialized diabetes clinic in the HNSM. In SSA, it is now well recognized that there is an important need for targeted training of specialists in the management of non-communicable diseases such as diabetes, and to counter the previous predominant focus on infectious diseases [14]. However, specialist training programs in SSA are lacking, and when available, they are not always well adapted to the local contexts and available resources, described by certain authors as being often Eurocentric [15,16]. In order to address this issue, both external and local trainers co-created and jointly delivered the training curriculum, which was identified as one of the successes of this project and appreciated by the participants according to their feedback. In addition to the contextual adaptation of the training curriculum, key elements to ensure the success and sustainability that have been identified in other similar training programs include on-site training by external faculty, shared country financial responsibility for the training, and transfer of training responsibility and capacity building to the local faculty, elements that are currently being discussed in the next steps of this project [17,18].

Regarding the interdisciplinary team that was trained, it was composed of physicians, nurses and nutritionists. The implementation of an interdisciplinary team approach to diabetes management is recommended and demonstrated to help PWD achieve their glycaemic goal [19,20]. Unfortunately, the nutritionists were quickly unavailable, one due to migration to another country seeking better working opportunities. Indeed, the retention of qualified personnel is another issue that healthcare systems in SSA have to face [14]. According to a recent review, the retention of HCPs in SSA is influenced by location of training as well as career prospects and employment opportunities after graduation [14]. In GB, a strategy is lacking for attracting professionals and retention of HCPs. In several African countries, strategic plans were developed to address this issue, including financial incentives particularly to recruit and retain health workers to rural and hard-to-reach areas [21]. Regarding the diabetes clinic, the purpose of establishing it within the HNSM was to integrate it into the existing health system and strengthen already available infrastructure. These are key elements to ensure sustainability that were also identified in other similar training programs in resource-constrained countries [17]. Several barriers leading to occasional interruptions of service were encountered, including a political instability in the country and strikes of healthcare staff demanding better wages and working conditions [22]. Health sector strikes related to demands of better remuneration are not uncommon in SSA [23]. To ensure successful running of such consultation clinics, continued buy-in and support from stakeholders should be ensured. We also recommend that diabetes training be incorporated in pre-and post-graduate training curriculums of all HCP to help shape a better workforce.

## Conclusion

This study delineates the feasibility of setting up a diabetes consultation clinic in GB despite important barriers. The problems encountered and the lessons learned from this project will hopefully serve to instruct those who may be considering starting their own diabetes training programs and clinics in developing countries.

### What is known about this topic

- Diabetes care requires an adequately trained interdisciplinary healthcare team;
- Diabetes training programs are lacking in Sub-Saharan Africa, including Guinea-Bissau.

### What this study adds

- This paper reports the implementation process of a diabetes training program and setting up of a specialized diabetes clinic in Guinea-Bissau;
- The evaluation of activities is presented and recommendations put forward to help guide other training programs in the future.
